# Familial hypomagnesaemia, Hypercalciuria and Nephrocalcinosis associated with a novel mutation of the highly conserved leucine residue 116 of Claudin 16 in a Chinese patient with a delayed diagnosis: a case report

**DOI:** 10.1186/s12882-018-0979-1

**Published:** 2018-07-13

**Authors:** Jingru Lu, Xiangzhong Zhao, Alessandro Paiardini, Yanhua Lang, Irene Bottillo, Leping Shao

**Affiliations:** 1 Department of Nephrology, The Affiliated Hospital of Qingdao University, 16 Jiangsu Road, Qingdao, 266003 China; 2 Central Laboratory, The Affiliated Hospital of Qingdao University, 1677 Wutaishan Road, Qingdao, 266555 China; 3 Dipartimento di Scienze Biochimiche “A. Rossi Fanelli”, Sapienza - Università di Roma, 00185 Rome, Italy; 4 Division of Medical Genetics, Department of Molecular Medicine, Sapienza University, San Camillo-Forlanini Hospital, Rome, Italy

**Keywords:** Familial hypomagnesaemia with Hypercalciuria and Nephrocalcinosis, Claudin 16, Mutation, Highly conserved motif

## Abstract

**Background:**

Sixty mutations of claudin 16 coding gene have been reported in familial hypomagnesemia with hypercalciuria and nephrocalcinosis (FHHNC) patients. Recent investigations revealed that a highly conserved glycine-leucine-tryptophan (^115^G-L-W^117^) motif in the first extracellular segment (ESC1) of claudin 16 might be essential for stabilization of the appropriately folded ECS1 structure and conservation of normal claudin 16 function. However, neither missense nor nonsense mutation has ever been described in this motif. Our study aimed at identifying mutations in a Chinese patient with FHHNC and exploring the association between genotype and phenotype.

**Case presentation:**

A 33-year-old female presented with 4 years history of recurrent acute pyelonephritis without other notable past medical history. Her healthy parents, who aged 56 and 53 respectively, were second cousins, and her only sibling died from renal failure without definite cause at age 25. Renal ultrasound imaging demonstrated atrophic kidneys and bilateral nephrocalcinosis. The laboratory workup revealed impaired renal function (Stage CKD IV), hypocalcemia and mild hypomagnesemia, accompanied with marked renal loss of magnesium and hypercalciuria. During the follow-up, treatment with calcitriol and calcium but not with magnesium was difficult to achieve normal serum calcium levels, whereas her serum magnesium concentration fluctuated within normal ranges. In the end, the patient unavoidably reached ESRD at 36 years old. The clinical features and family history suggested the diagnosis of FHHNC. To make a definite diagnosis, we use whole-exome sequencing to identify the disease-causing mutations and Sanger sequencing to confirm the mutation co-segregation in the family. As a result, a novel homozygous mutation (c.346C > G, p.Leu116Val) in ^115^G-L-W^117^ motif of claudin 16 was identified. Her parents, grandmother and one of her cousins carried heterozygous p.Leu116Val, whereas 200 unrelated controls did not carry this mutation.

**Conclusions:**

We described a delayed diagnosis patient with FHHNC in the Chinese population and identified a novel missense mutation in the highly conserved ^115^G-L-W^117^ motif of claudin 16 for the first time. According to the reported data and the information deduced from 3D modeling, we speculate that this mutation probably reserve partial residual function which might be related to the slight phenotype of the patient.

**Electronic supplementary material:**

The online version of this article (10.1186/s12882-018-0979-1) contains supplementary material, which is available to authorized users.

## Background

Familial hypomagnesemia with hypercalciuria and nephrocalcinosis (FHHNC) is a rare autosomal-recessive renal tubular disorder characterized by excessive urinary losses of magnesium and calcium, bilateral nephrocalcinosis and progressive chronic renal failure [1]. This disease is caused by mutations in the tight junction (TJ) proteins, claudin-16 and -19, which are encoded by the CLDN16 (OMIM #248250) or CLDN19 (OMIM #248190) genes respectively [2, 3]. These two proteins are expressed in the thick ascending limb (TAL) of Henle’s loop and are involved in the paracellular reabsorption of calcium and magnesium [4]. There is no evident difference in renal phenotypes between patients with mutations in CLDN16 and CLDN19, however, severe ocular involvement has been described in CLDN19 patients that might be attributed to extra-renal expression of claudin-19 in nervous system and retina [3].

So far, 60 different mutations of the CLDN16 gene have been described, including 39 missense mutations, 7 nonsense mutations, 6 splice site mutations, 4 small deletions and 4 other types of mutations (Additional file 1: Figure S1) [2, 5–25]. These mutations spread across the five exons of the CLDN16 gene, and most missense mutations are located in or near the four transmembrane domains, especially in the two extracellular segments (ECSs) and in the carboxy-terminal cytoplasmic region (Additional file 1: Figure S1). The majority of previously studied patients with FHHNC came from European, Middle Eastern and North African countries, and only a few had been reported in USA, East Asia or South Asia [7]. So far only a homozygous CLDN16 indel mutation associated with FHHNC has been reported in two patients from a Chinese family [22].

Research studies using mouse and cell models have generated significant advances in the understanding of the pathophysiology of FHHNC. The determination of the first crystal structure of a mammalian claudin (claudin-15) would not only provide structural information on this family of TJ proteins but also help to further understand the molecular mechanisms of this disease [25]. In the first extracellular segment (ESC1), a highly conserved glycine-leucine-tryptophan (^115^G-L-W^117^) motif among all members of the claudin family has been found probably helpful for stabilization of the appropriately folded ECS1 structure [26]. However, no missense mutation was reported in these three amino acid residues thus far. Here, we reported a Chinese FHHNC family associated with a novel missense mutation of the highly conserved Leucine residue 116 in the ^115^G-L-W^117^ motif.

## Case presentation

**Fig. 1:**
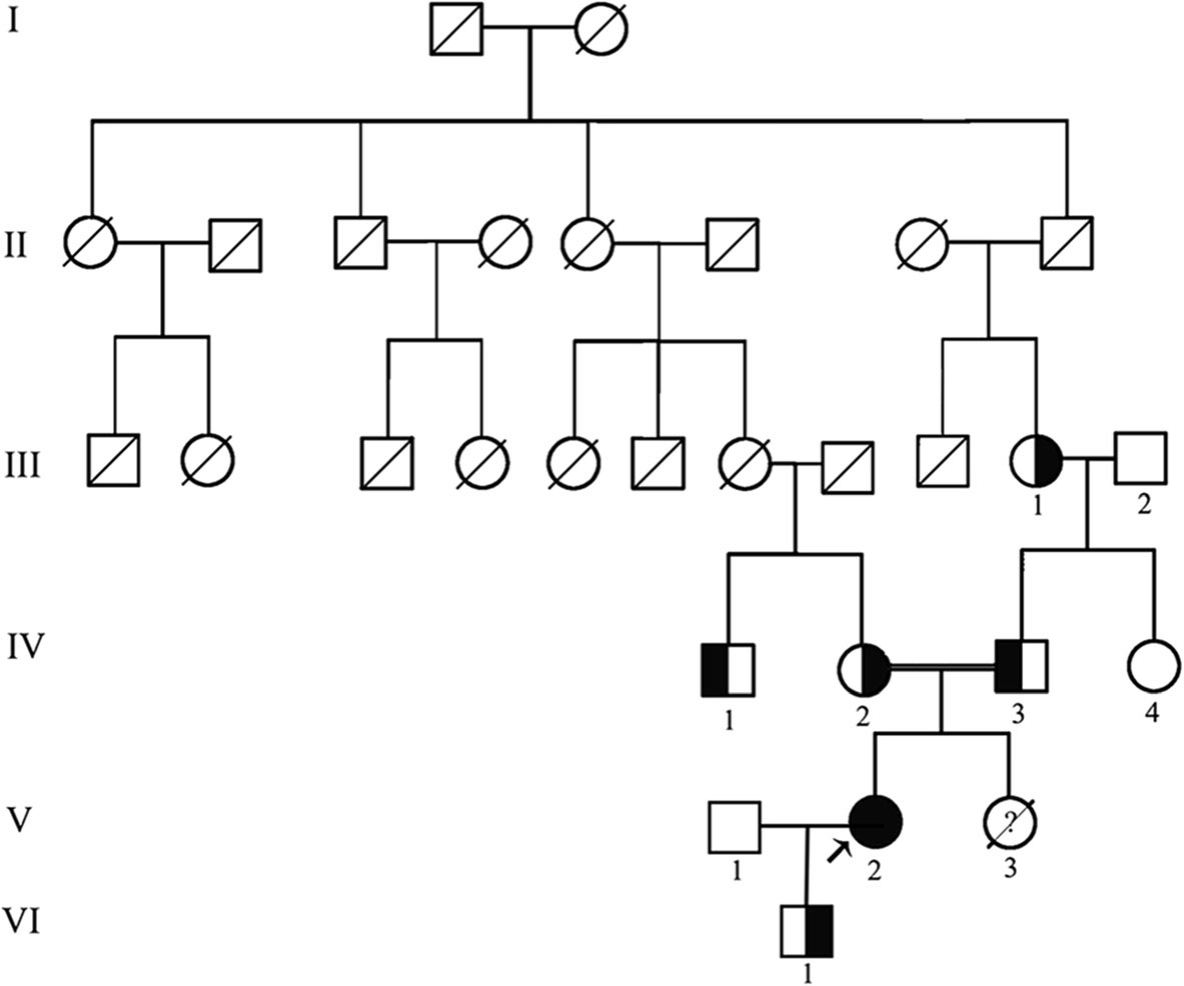
Pedigree of the Chinese family with familial hypomagnesemia with hypercalciuria and nephrocalcinosis. □, male; ○, female; ↗, proband

**Fig. 2:**
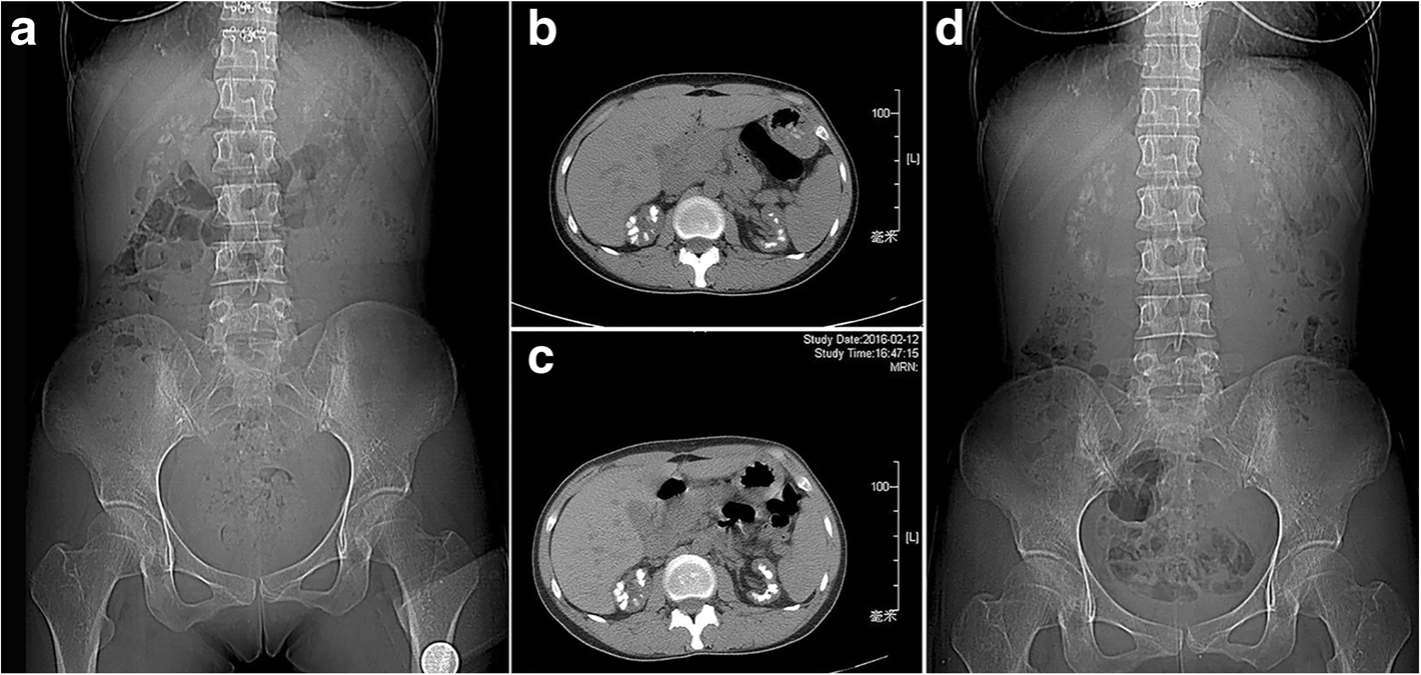
Imaging of renal tract in the proband. **a**. Plain abdominal radiograph showing bilateral nephrocalcinosis, aged 34 years. **b**. CT scan showing bilateral nephrocalcinosis, aged 34 years. **c**. CT scan showing bilateral nephrocalcinosis, aged 36 years. **d**. Plain abdominal radiograph showing bilateral nephrocalcinosis, aged 36 years

In Mar 2013, a 33-year-old female came to the nephrology department because of 4 years of recurrent acute pyelonephritis. She had no other notable past medical history including polyuria, polydipsia, muscular cramps, carpopedal spasms or generalized seizures, and did not take any regular medication. Her parents, who aged 56 and 53 respectively, were second cousins and denied any remarkable medical history. Her only sibling died from renal failure without definite cause at age 25. The pedigree of the family is shown in Fig. 1. Physical examination revealed that her height was 160 cm (The average height of Chinese adult females at corresponding age is 159 cm.) and her weight was 55 kg with a BMI of 21.48 kg/m^2^. Laboratory workup revealed impaired renal function (SCr 250 μmol/L, EPI-eGFR = 21.1 ml/min/1.73m^2^), hypocalcemia (1.42 mmol/l, normal range 2.11–2.52), and normal serum parathyroid hormone levels (65.59 pg/ml) in the context of normal 25OH-Vitamin D levels (26 ng/ml, reference range 20.0–32.0) (Table 1). Her serum magnesium level was slightly low (0.60 mmol/l, reference range 0.65–1.20), and 24-h urinary calcium was 3.9 mmol/1.73m^2^ (normal range 2.5–5.5) in the setting of decreased renal function. Distal renal tubular acidosis was excluded since she had normal urine acidification function (pH < 5.3) in the setting of nearly normal serum bicarbonate level (HCO_3_^−^ 22 mmol/l). Renal ultrasound imaging demonstrated bilateral nephrocalcinosis and parenchymal renal calculi, with the right kidney length 9.5 cm and the left 9.4 cm. Ophthalmologic examination was normal. During the subsequent 3 years of follow-up, she had undergone six attacks of acute UPI. In the interictal phase of infections, she had accepted four times of biochemical assessment (Table 1). Repeated examinations in the follow-up revealed marked renal loss of magnesium (fractional excretion 42.7 ± 7.4%, normal range less than 5%) and hypercalciuria (urinary calcium/creatine 0.52 ± 0.08 mol/mol, normal range 0.15–0.33). Treatment with calcitriol and calcium but not with magnesium was difficult to achieve a considerable effect on her serum calcium, whereas her serum magnesium concentration fluctuated within the normal range (0.70–0.91 mmol/l) under the circumstances of renal failure. Plain abdominal radiograph and abdominal CT scanning, which were performed in Mar 2014 and Feb 2016 respectively, demonstrated gradually aggravated nephrocalcinosis and atrophy of renal parenchyma (Fig. 2). In the end, the patient unavoidably reached ESRD at the age of 36. The gradient of GFR decline was calculated to be 4.3 ml/min per 1.73 m^2^/yr. during the follow up, whereas the gradient of GFR decline was about 2.1 ml/min per 1.73 m^2^/yr. 3 years before the period of follow up, and the approved global slope of decline was 2.5 ml/min per 1.73 m^2^/yr.

**Table 1:** Laboratory findings of the proband during the hospitalization and 3-year follow-up

Items	First Admission	First follow-up	Second follow-up	Third follow-up	Fourth follow-up	Normal Range
Date	Mar, 2013	Mar, 2014	Aug, 2014	Feb, 2016	Jun, 2016	
Age (yrs)	33	34	34	36	36	
SCr (μmol/L)	250	270	306	537	647	31–132
eGFR^a^ (ml/min/1.73m^2^)	21.1	19.1	16.4	8.2	6.5	> 90
SK (mmol/L)	4.2	4.3	4.4	4.9	4.7	3.5–5.5
SMg (mmol/L)	0.65	NA	0.70	0.99	0.91	0.65–1.20
SCa (mmol/L)	1.42	1.63	1.68	1.81	1.58	2.11–2.52
SP (mmol/L)	1.7	2.0	1.5	2.0	2.1	0.8–1.6
HCO_3_ (mmol/L)	22.0	21.0	20.0	23.7	21.7	23.0–31.0
ALB (g/L)	37.2	NA	47.8	39.6	46.5	35.0–55.0
ALP (U/L)	40.8	NA	NA	NA	60.9	35–125
PTH (pg/ml)	65.6	74.5	75.6	73.8	94.5	15.0–65.0
UpH	5.0	5.0	NA	NA	NA	
24 h-UCa (mmol/1.73m^2^)	3.9	4.3	3.8	2.1	2.0	2.5–5.5
UCa/Cr (mol/mol)	NA	0.61	0.57	0.46	0.44	0.15–0.33
24 h-UMg (mmol/1.73m^2^)	NA	7.1	7.0	3.9	4.2	
FEMg^b^	NA	38.5%	43.7%	35.9%	52.7%	< 5.0%
25OH-VitD	NA	NA	NA	NA	22.4	20.0—32.0
UProtein	1.07 g/24 h	+	NA	NA	+	Negative

*eGFR* Estimated glomerular filtration rate, *S* Serum, *ALB* Albumin, *ALP* Alkaline phosphate, *PTH* Parathyroid hormone, *U* Urine, *NA* Not available. a, Calculated by CKD-EPI formula; b, Calculated by the following formula: UMg × SCr/(0.7 × SMg × UCr) × 100%

**Fig. 3:**
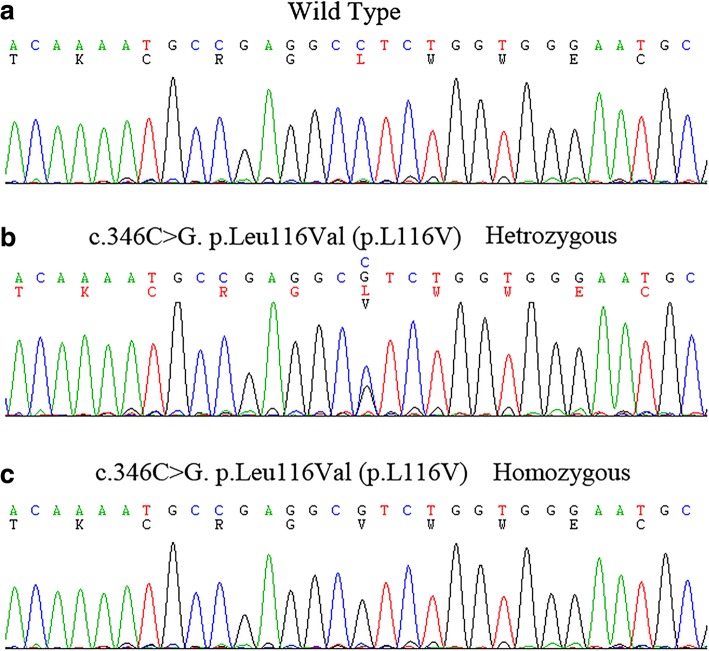
The novel missense mutation (c.346C > G, p.Leu116Val) identified in the patient with familial hypomagnesemia with hypercalciuria and nephrocalcinosis. **a**. Wild type; **b**. Heterozygous mutation of p.Leu116Val; **c**. Homozygous mutation of p.Leu116Val

Nephrocalcinosis associated with end-stage renal failure is usually seen in three genetic diseases: primary hyperoxaluria, Dent’s disease, and FHHNC. Meanwhile, hypercalciuric hypomagnesemia is mainly seen in FHHNC, autosomal dominant hypocalcemia with hypercalciuria (CASR gain-of-function), or Bartter syndrome type III. Although the patient’s family history, hypocalcemia, and hypomagnesemia suggested FHHNC, we performed whole-exome sequencing (WES) to exclude the possibilities of comorbid diseases or potential mutations of other genes that could affect the renal reabsorption of calcium and magnesium. All the family members and healthy controls gave informed consent. The study protocol was approved by the Ethics Committee on Human Studies at the Affiliated Hospital of Qingdao University. As a result, a homozygous cytosine-to-guanine substitution at position 346 of the open reading frame (c.346C > G) in exon 2 of CLDN16 gene was identified by WES. This single nucleotide alteration led to a single amino acid substitution from leucine to valine at amino acid position 116 of claudin 16 (p.Leu116Val; Of note, there is still controversy about the physiological use of the initiation codon. The numbering of the mutation regarded to the second ATG would be p.Leu46Val.) (Fig. 3). Sanger sequencing validation of all family members revealed that four of her unaffected members including her parents, grandmother and one of her cousins carried heterozygous p.Leu116Val mutation in CLDN16, whereas other family members and 200 unrelated controls from the same ethnic background (Chinese Han population) did not carry this mutation. This novel variant was highly conserved among 8 different species (Human, Rat, Frog, Chimpanzee, Eagle, Horse, Turtle & Cattle) (Additional file 2: Figure S2) and among all 27 Human Claudins family members, and never been reported in 1000G, ExAC, or EVS. All four in silico prediction tools (SIFT, PolyPhen2, Mutation taster, and PROVEAN) showed a possibility of disease-causing for FHHNC. In addition, analysis by HSF 3.0 (http://www.umd.be/HSF3/HSF.shtml) software showed that no significant alteration of splicing regulatory elements occur after mutation.

**Fig. 4:**
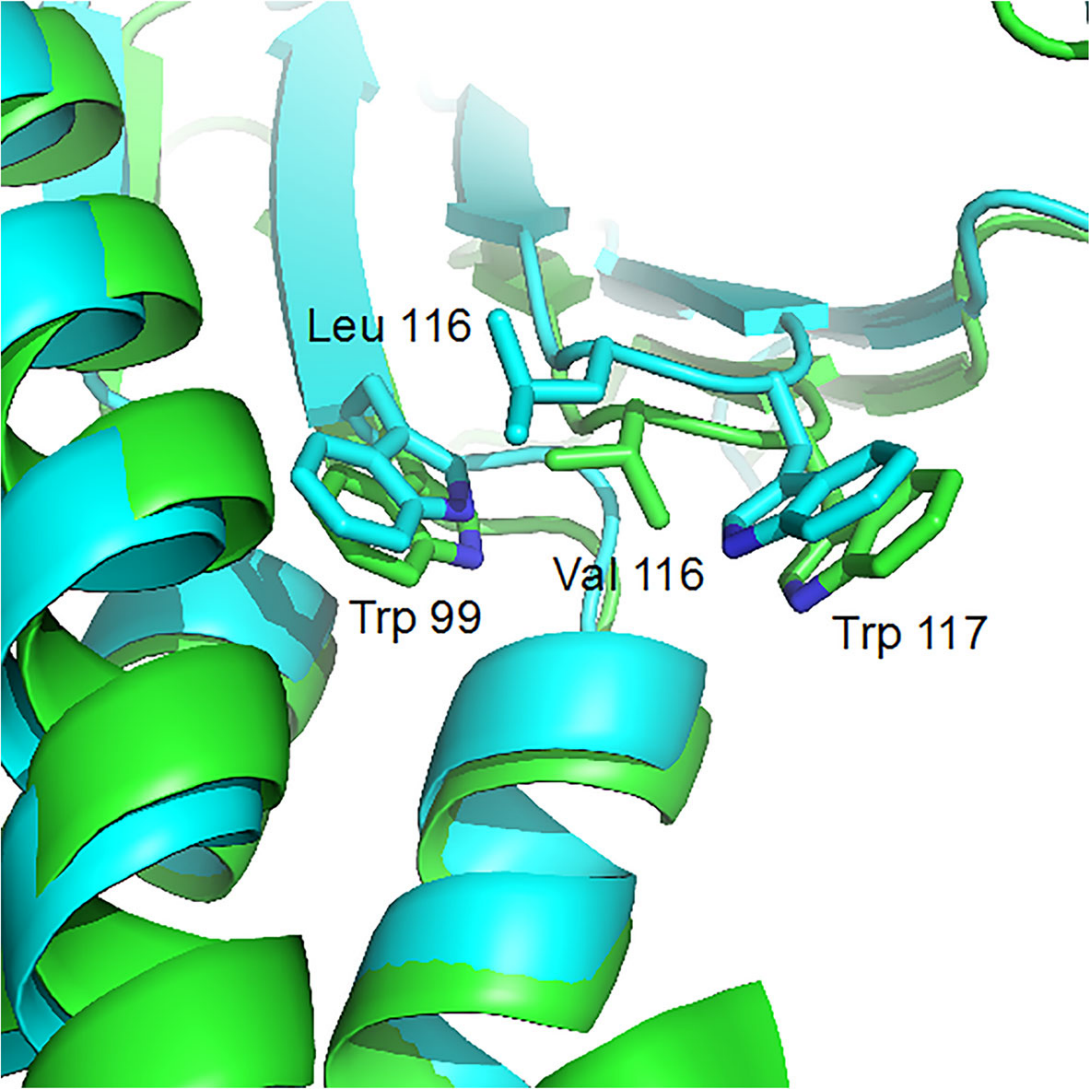
Comparison between Wild-Type Claudin 16 (cyan ribbons) and L116 V mutant (green ribbons). The conserved W/LW motif of the Claudin family (Trp99, Leu116, and Trp117 in Claudin 16) is shown as sticks

A three-dimensional (3D) model of Claudin 16 was built using the homology modeling approach implemented in the modeler-9 package. The result showed that the conserved W-L-W motif of the Claudin family (Trp99, Leu116, and Trp117 in Claudin 16) was embedded in a crevice formed by the top of the four-helix bundle. Leu116 and Trp117 protrude from the tip of the β2-β3 loop and were likely associated with Trp99 to serve as a “hydrophobic anchor” for the β-sheet domain. The L116 V mutation was predicted to abolish such interaction, resulting in a displacement of the β-sheet domain from the four-helix bundle domain of Claudin 16 (Fig. 4).

## Discussion and conclusions

Since the first clinical description of FHHNC by Michelis et al. in 1972 [27], only about 120 cases were reported worldwide. In the Chinese population, only two genetically confirmed FHHNC cases from a consanguineous family were described so far, and a homozygous indel mutation (c.574_589delinsTACCGGTCTGGCTGGACTAGCAA) was found [22]. In this paper, we described the phenotype of a FHHNC patient from another family and find a novel missense mutation (c.346C > G, p.Leu116Val) by WES.

The pathogenesis of chronic kidney disease (CKD) in FHHNC remains unclear. In the period of 3 years of follow-up, the patient exhibited a faster decline in renal function compared with the early phase of before 33 years of age, this may be related to the renal interstitial nephritis and accelerated renal parenchyma damage due to UPI, and the inflammation caused by the infection may further promote calcium deposition in the kidney [28]. Therefore, the nephrocalcinosis and infection played a significant negative impact on the chronic kidney disease (CKD). However, apparently, claudin-16 might actively involve the impairment of renal function through still unclear mechanisms, since not all inherited tubulopathies characterized by nephrocalcinosis do uniformly lead to ESRD. The insidious course of the patient’s sister suggested a potential role of claudin 16 to some extent. Recently, it is speculated that Claudin 16 not only is the major constituent of TJ and forms strands that mediate cell adhesion and function as paracellular barriers in the TAL of Henle’s loop, but also may be involved in the regulation of cell growth, proliferation, differentiation, and dedifferentiation [29, 30]. The defective claudin-16 probably leads to the disruption of tubular TJ complex, and subsequent alterations of cell polarity and an induction of tubular dysplasia, and resultant transforming of tubular epithelial cells into new fibroblasts that was well acknowledged as a progress of renal fibrosis and renal failure.

Konrad et al. had established a phenotype-genotype correlation regarding renal failure progression in FHHNC patients with CLDN16 mutations [17]. They had studied a cohort of 71 affected individuals and found that patients with complete loss of function mutations in both alleles exhibit a younger age at the presentation of FHHNC symptoms (2.2 versus 5.6 yrs) and a more rapid decline in renal function (7.3 versus 2.9 ml/min per 1.72 m^2^/yr) when compared with those bearing at least one mutation reserving partial function [17]. These data suggested that the presence of at least one *CLDN16* allele with partial function was enough to predict a relatively milder clinical phenotype. Accordingly, the proband with a mild phenotype reflected that the mutation p.Leu116Val probably retained a significant residual claudin-16 function.

Recently Suzuki et al. reported the first crystal structure of a mammalian claudin (claudin-15), providing structural information on this family of TJ proteins [26]. Claudin 16 consists of four transmembrane domains (TM1–4), two extracellular segments (ECS1 and ECS2), amino- and carboxy-terminal cytoplasmic tails and a short cytoplasmic loop. The TM segments form a typical left-handed four-helix bundle, and large portions of the two ECSs form a prominent β-sheet structure [26]. The β-sheet domain extends from the membrane surface and comprises five β strands (β1 to β5), four contributed by ECS1 and one by ECS2. At the end of ECS1, the β4 strand connects to a short extracellular helix (ECH) that connects to TM2 after a sharp bend. Of note, ECS1 contains a conserved ^115^G-L-W^117^ motif that seems to contribute to the stabilization of the appropriately folded ECS1 structure [26]. According to the 3D model, Leu116 and Trp117 protrude from the tip of the β2-β3 loop and appear to be close to the extracellular membrane surface, serving as an “anchor” for the β-sheet domain. In addition, Leu116, via its main-chain carbonyl groups, forms hydrogen bonds with the guanidinium group of Arg149, further stabilizing the β-sheet domain and the following ECH region [26]. Therefore, we proposed that mutation p.Leu116Val may lead to instability of the extracellular domains of the membrane protein of claudin 16, and accordingly the reduction of the intrinsic activity. Most noteworthy, the investigation regarding claudin 1 demonstrated that alanine substitution of the residue Leu50 (corresponding to Leu116 of claudin 16) did not impair claudin-1 cell surface expression or lateral protein interactions within the plasma membrane, however, on the contrary, disturb the formation of cell-cell contacts and hepatitis C virus entry [31]. Combining all the data from above-mentioned investigations regarding claudin 1, and genotype-phenotype associations of FHHNC, it was reasonable to presume that mutant claudin 16 with p.Leu116Val, although with a significant loss of function compared with wild-type claudin-16, probably could properly target to the cell membrane and TJ, and still retain a significant residual function. However, the exact molecular pathogenetic mechanisms of the mutation need further in vitro expression study to confirm.

There are three possible reasons why proband have not been diagnosed in the past 4 years. Firstly, although the proband had nephrocalcinosis, noticeable hypomagnesemia was absent. She had no other notable past medical history including polyuria, polydipsia, muscular cramps, carpopedal spasms or generalized seizures, and did not take any regular medication. It is easy to understand that patients without typical clinical manifestations were not easily diagnosed. Secondly, only two cases were reported in China, and it is common that Clinicians lacked understanding of the disease. Lastly, the proband had not been evaluated after the death of her sibling because of kidney failure of unknown origin.

Distal renal tubular acidosis (dRTA) is common in FHHNC patients. Weber S et al. found that 17 in 20 patients with CLDN16 mutations presented with incomplete dRTA [6]. The distal acidification defect was due both to defective ammonia transfer to the distal nephron and to defective hydrogen ion secretion at the level of the medullary collecting duct [32]. Since this defect is probably secondary to the medullary interstitial nephropathy caused by FHHNC, dRTA would not inevitably appear in some patients, just like our patient. Rickets and/or pathological fractures, probably caused by chronic hypocalcemia, metabolic acidosis, secondary hyperparathyroidism, were not commonly reported in FHHNC [20] and were absent in this patient. Moreover, noticeable hypomagnesemia was also absent in our patient, which possibly related to the decreased GFR leading to lower filtration of magnesium. In the background of renal insufficiency, fractional excretion of magnesium might be especially helpful for timely diagnosis.

In summary, we identified a novel missense mutation (c.346C > G, p.Leu116Val) in the highly conserved ^115^G-L-W^117^ motif of claudin 16 for the first time. Based on previous investigations and 3D modeling of claudin 16, this mutation was proposed to lead to instability of the ECS1 domains, but probably preserves significant residual function, which could be further proved by the mild clinical phenotype resulted from this novel mutation seen in our patient. Meanwhile, the exploration of the molecular pathogenic mechanisms of this missense mutation will particularly be interesting.

## Additional files

Additional file 1: **Figure S1.** Schematic representation of the claudin-16 protein and positioning of reported missense/nonsense mutations in CLDN16. The underlined is the novel mutation p.L116 V identified in this study. On the basis of the results of mutation analysis and sequence comparisons, the claudin 16 protein seems to be shorter than reported previously (the 70 amino-terminal amino acids that are presumably lacking are depicted in gray). (PNG 21796 kb)

Additional file 2: **Figure S2.** The result of sequence alignment on 8 species of Claudin 16 homologous proteins. Black arrows are pointing to leucine at position 116. (TIF 2004 kb)

## Abbreviations

^115^G-L-W^117^: glycine-leucine-tryptophan motif
BMI: Body Mass Index
CKD: Chronic kidney disease
ECSs: Extracellular segments
ESRD: End-stage renal disease
FHHNC: Familial hypomagnesemia with hypercalciuria and nephrocalcinosis
GFR: Glomerular filtration rate
PCR: Polymerase chain reaction
TAL: Thick ascending limb
TJ: tight junction
TM: Transmembrane domains
UPI: The upper urinary tract infection
