# Supplementary figures and images for: Familial hypomagnesaemia, Hypercalciuria and Nephrocalcinosis associated with a novel mutation of the highly conserved leucine residue 116 of Claudin 16 in a Chinese patient with a delayed diagnosis: a case report
Source: BMC Nephrol. 2018 Jul 13;19:181. doi: 10.1186/s12882-018-0979-1 (PMC6045852; doi:10.1186/s12882-018-0979-1)

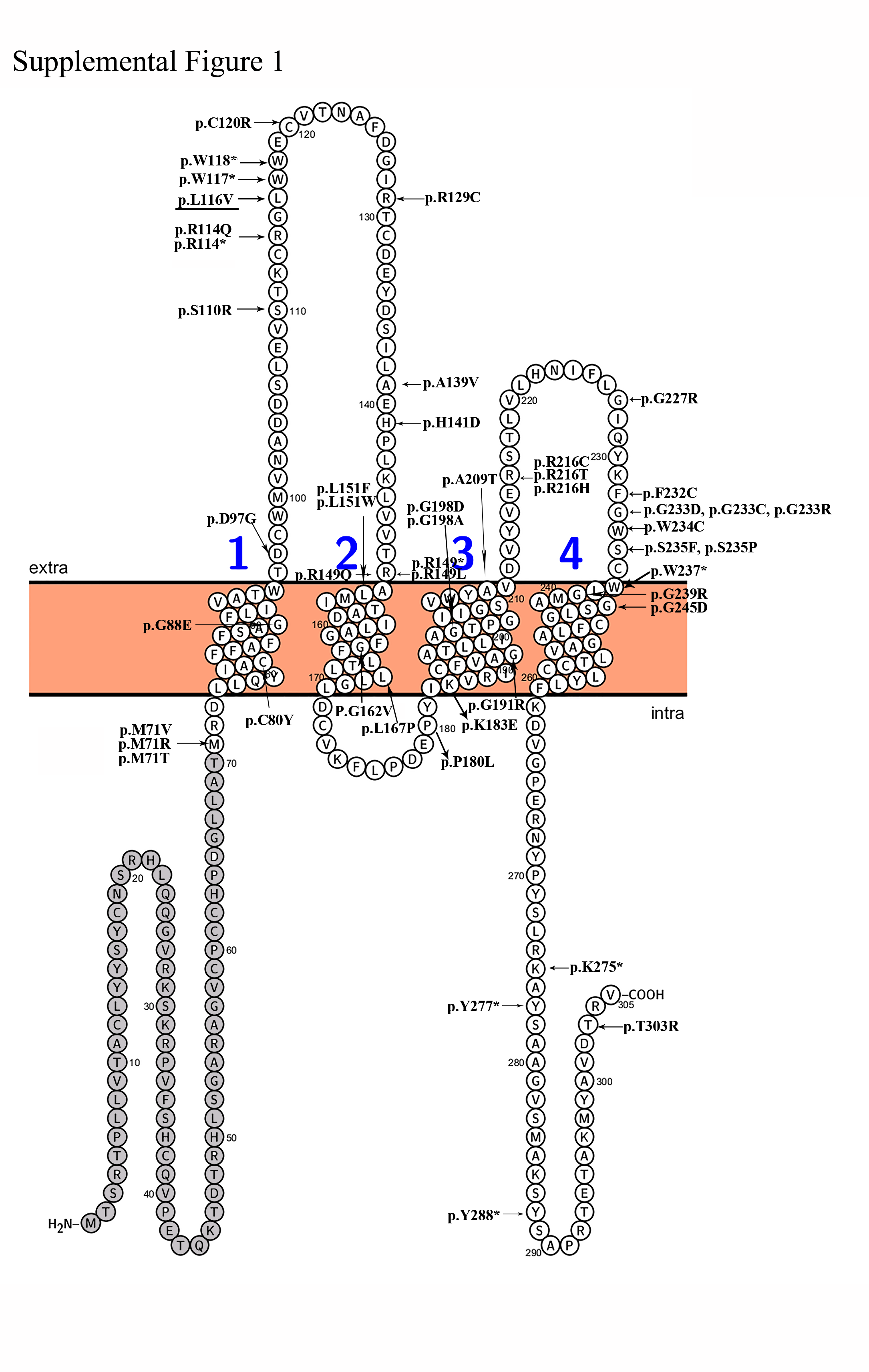

Supplement: Supplementary file 1 — Figure S1. Schematic representation of the claudin-16 protein and positioning of reported missense/nonsense mutations in CLDN16. The underlined is the novel mutation p.L116 V identified in this study. On the basis of the results of mutation analysis and sequence comparisons, the claudin 16 protein seems to be shorter than reported previously (the 70 amino-terminal amino acids that are presumably lacking are depicted in gray). (PNG 21796 kb) [file 12882_2018_979_MOESM1_ESM.png]

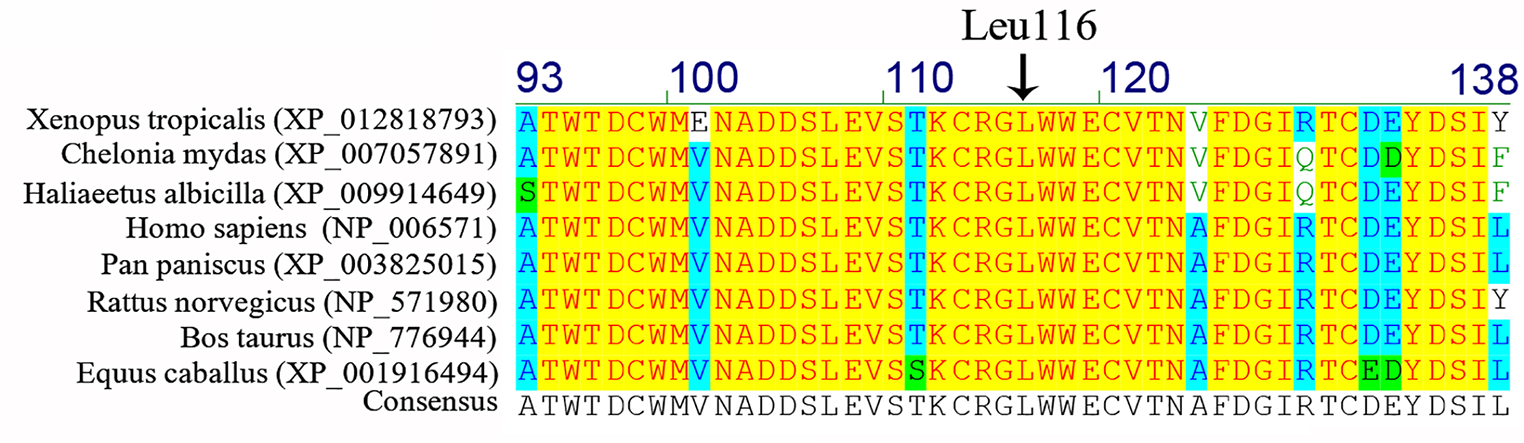

Supplement: Supplementary file 2 — Figure S2. The result of sequence alignment on 8 species of Claudin 16 homologous proteins. Black arrows are pointing to leucine at position 116. (TIF 2004 kb) [file 12882_2018_979_MOESM2_ESM.tif]
